# Surveillance of Tick-Borne Pathogens in Ticks from Humans in the Province of Verona, Italy (2018–2022): A Prospective Study

**DOI:** 10.3390/microorganisms13050965

**Published:** 2025-04-23

**Authors:** Lucia Moro, Graziana Da Rold, Anna Beltrame, Fabio Formenti, Cristina Mazzi, Andrea Ragusa, Salvatore Scarso, Ilenia Drigo, Monica Degani, Chiara Piubelli, Carlo Vittorio Citterio, Francesca Perandin

**Affiliations:** 1 Department of Infectious, Tropical Diseases and Microbiology, IRCCS Sacro Cuore Don Calabria Hospital, 37024 Negrar di Valpolicella, VR, Italy; lucia.moro@sacrocuore.it (L.M.); anna.beltrame@moffitt.org (A.B.); fabio.formenti@sacrocuore.it (F.F.); andrea.ragusa@sacrocuore.it (A.R.); salvatore.scarso@sacrocuore.it (S.S.); monica.degani@sacrocuore.it (M.D.); francesca.perandin@sacrocuore.it (F.P.); 2 Istituto Zooprofilattico Sperimentale delle Venezie (IZSVe), SCT2-Belluno, 32100 Belluno, VR, Italy; gdarold@izsvenezie.it (G.D.R.); idrigo@izsvenezie.it (I.D.); ccitterio@izsvenezie.it (C.V.C.); 3 Clinical Research Unit, IRCCS Sacro Cuore Don Calabria Hospital, 37024 Negrar di Valpolicella, VR, Italy; cristina.mazzi@sacrocuore.it

**Keywords:** zoonosis, human infection, *Borrelia*, *Rickettsia*, *Anaplasma*, Babesia, TBE virus, Northern Italy

## Abstract

In Italy, the epidemiology of tick-borne pathogens is still poorly characterized. This prospective study was conducted at the IRCCS Sacro Cuore Don Calabria Hospital in Negrar di Valpolicella (Verona), northeastern Italy, from 2018 to 2022. Ticks from asymptomatic individuals visiting the hospital after a recent tick bite were characterized using microscopy and tested for pathogens using molecular tests. A total of 317 ticks collected from 280 subjects were analyzed, with most identified as *Ixodes* species (95.6%), followed by *Rhipicephalus* spp. (0.6%) and *Dermacentor* spp. (0.3%). Molecular analysis was performed on 257 single ticks and 23 pooled samples. Overall, 15.4% tested positive for at least one pathogen. The most frequently detected pathogen was *Borrelia* spp. (n = 22, 7.8%), including *B. afzeli* (n = 8), *B. miyamotoi* (n = 6), *B. valaisiana* (n = 2), *B. garinii* (n = 2), *Borrelia* spp. (n = 2), *B. burgdorferi sensu stricto* (n = 1), and *B. spielmanii* (n = 1). *Rickettsia* spp. was detected in 20 samples (7.1%), comprising *R. helvetica* (n = 11), *R. monacensis* (n = 7), and *Rickettsia* spp. (n = 2). Other pathogens included *Anaplasma phagocytophilum* (n = 5, 1.8%), *Babesia venatorum* (n = 2, 0.7%), and tick-borne encephalitis virus (n = 1, 0.4%). This study calls for enhanced surveillance in the province of Verona to clarify these pathogens’ clinical impact.

## 1. Introduction

In recent decades, Europe has seen a marked increase in the incidence of tick-borne diseases (TBDs), even in areas not previously considered endemic [1]. This increase is driven by general factors, such as climate changes and fluctuation in wildlife population abundance and dynamics [2], as well as other local, specific risk factors [3,4,5]. Furthermore, a significant presence of ticks infected with various tick-borne pathogens (TBPs) has been observed in European urban and peri-urban green areas, posing a risk to human and animal health due to the high exposure in these environments [6].

In Italy, the distribution of TBPs varies between the north and south, influenced by ecological, climatic, and human factors [7]. However, epidemiological data on human TBDs remain limited [7,8,9,10]. The absence of mandatory reporting and the scarcity of diagnostic tests likely contribute to a significant underestimation of their true incidence. Existing studies are often fragmentary, further complicating the understanding of TBD epidemiology in the region [11,12].

A rich diversity of tick species, including *Ixodes ricinus (I. ricinus)*, *Haemaphysalis inermis*, *Rhiphicephalus turanicus*, *Dermacentor marginatus*, and *Hyalomma marginatum*, is observed in southern Italy and the islands [7,13]. In these regions, Mediterranean spotted fever (MSF), caused by *Rickettsia conorii*, is the predominant rickettsial disease, although other *Rickettsia (R.)* subspecies with varying pathogenicity have also been identified in ticks [7,14].

In contrast, in Northern and Central Italy, *I. ricinus* is the dominant tick species, and Lyme borreliosis (LB) is the most common TBD [15,16]. LB is mainly caused by *Borrelia (B.) burgdorferi sensu lato* complex, including *B. burgdorferi sensu stricto*, *B. afzelii*, and *B. garinii*. LB is also caused by other species of *Borrelia,* such as *B. spielmanii* [17] and *B. valaisiana* [18], although their clinical presentation remains less well-defined.

In the last decades, an increased incidence of LB cases has been described in Italy [19], including the province of Verona [16]. Moreover, tick-borne encephalitis (TBE) caused by the tick-borne encephalitis virus (TBEV) (European subtype) has also shown an increasing incidence in northeastern Italy and has spread to areas not previously considered at risk [20,21]. In addition, a notable presence of *Rickettsia* species (*R. monacensis* and *R. helvetica*) and *Anaplasma (A.) phagogytophilum* has been reported on *I. ricinus* ticks in this region [7,8,9,15].

In contrast, little is known regarding possible emerging new pathogens in humans, such as *R. slovaca* [14], which causes tick-borne lymphadenopathy (TIBOLA), *B. miyamotoi*, responsible for relapsing fever [22]; *Babesia* spp., which causes a febrile syndrome, the severity of which varies greatly depending on the virulence of the strain and/or species and the age and immunological status of the host [23]; *Neoehrlichia mikurenisis*, an emerging pathogen that causes fever primarily in immunocompromised subjects [24].

The identification of TBPs from ticks removed from humans is increasingly being used to monitor changes in ticks and pathogens distributions [25]. Particularly with the rise of citizen science, when members of the public collaborate with scientists to collect data and samples, this approach provides a valid, cost-effective method for gathering information on the circulation of TBPs and estimating their real risk to humans [25]. Additionally, compared to tick dragging or tick flagging, this method allows for a more accurate estimation of the risk of contracting a pathogen through tick bites, both in traditional risk areas (such as forests) and in less commonly analyzed areas, such as urban parks or private gardens.

A preliminary study by Beltrame et al. in Veneto in 2016 analyzed 45 ticks removed from humans and detected *Rickettsia* spp. in 9%, *Borrelia* spp. in 7%, and *A. phagocytophilum* in 2% of samples from Verona and surrounding provinces [15]. Building on these findings, the current study aimed to provide a more comprehensive epidemiological assessment of TBPs that can be detected in ticks collected from humans, increasing the sample size and evaluating the occurrence of additional TBPs to inform public health strategies.

## 2. Materials and Methods

This prospective study was conducted at the IRCCS Sacro Cuore Don Calabria Hospital between 1 January 2018 and 31 December 2022. Individuals seeking medical attention due to a recent tick bite, either at the emergency department or a dedicated outpatient clinic, were invited to participate. They were asked to specify the likely geographic location of exposure to the tick bite.

Ticks were promptly identified by stereomicroscopy in the hospital’s parasitology laboratory and subsequently stored at −80 °C in Tropica Biobank (BBMRI-eric ID IT_1605519998080235). Molecular analysis was conducted at the Istituto Zooprofilattico Sperimentale delle Venezie (IZSVe) in Legnaro (Padova, Italy). Data were analyzed quantitatively using absolute and percentage frequencies.

Statistical analyses were performed using R software v4.2.3 (R Core Team, Vienna, Austria), and choropleth maps were generated using the sf package and ggplot2 [26,27]. The geographical map of the Veneto region was downloaded from https://gisportal.istat.it/IstatViewer/ (last accessed on 20 March 2025) and put together with the choropleth maps using the GIMP (GNU Image Manipulation Program) tool, version 2.10.38.

### 2.1. Tick Identification

All collected ticks were identified to genus level, life stage (i.e., larva, nymph, or adult), and gender (female or male) under a stereomicroscope using morphological keys appropriate for each developmental stage [28,29]. All ticks were stored at −80 °C in sample vials until molecular analysis. An individual sample number (ID) was assigned to vials. For subjects who provided only one tick, the laboratory proceeded with nucleic acid extraction for each tick. For subjects who provided more than one tick at a time, the laboratory proceeded to combine ticks of the same species into a single pool (regardless of stage) for each individual and subjected them to DNA extraction [30,31].

### 2.2. DNA Extraction

Single and pooled ticks were homogenized in 600 µL of Phosphate Saline Buffer (PBS) with two 3 mm beads (Qiagen, Hilden, Germany) using the instrument TissueLyser II (Qiagen). Then, 200 µL of homogenate was used for nucleic acids extraction using MagMAX™ Pathogen RNA/DNA Kit (Applied Biosystems™, Foster, CA, USA). DNA/RNA extraction was performed using the Hamilton Microlab STAR liquid handler (Hamilton Company, Reno, NV, USA). Elution was performed in 90 µL of the Elution buffer and stored at −80 °C until use.

### 2.3. Polymerase Chain Reaction (PCR) and Sequencing

The presence of *Borrelia* and the involved genospecies (*B. miyamotoi*, *B. afzelii*, *B. burgdorferi sensu stricto*, *B. garinii*) was investigated using specific TaqMan qPCR assays (Supplementary Table S1) [32,33,34]. The samples positive for *Borrelia* spp. in real time but not belonging to any of the genospecies screened with specific probes (*B. miyamotoi*, *B. afzelii*, *B. burgdorferi sensu stricto*, *B. garinii*), were amplified using end-point one-step PCR and sequenced (Supplementary Table S1) by Sanger sequencing [35]. DNA was screened for the detection of *Rickettsia* spp. and *Babesia* spp. using end-point one-step PCR and Sanger sequencing and for *Anaplasma phagocytophilum* using a real-time assay with a specific probe (Supplementary Table S1) [32,36,37]. In detail, each qPCR reaction was carried out in a total volume of 20 µL using QuantiNova Probe PCR Kit (Qiagen) and the amplifications were performed in a CFX96 Touch Real-Time PCR Detection System (BioRad, Milan, Italy) using an initial denaturation step at 95 °C for 2 min, followed by 40 cycles consisting of denaturation at 95 °C for 5 s, annealing and elongation at 60 °C for 30 s. End-point PCR reactions were performed using AmpliTaq Gold™ DNA Polymerase with Buffer II and MgCl2 (Applied Biosystem™) in Veriti 96-well Thermal cycler (Applied Biosystems™). PCR products were analyzed by agarose (2% with SYBR Safe) gel electrophoresis using Invitrogen E-Gel Power Snap Plus Electrophoresis Systems (Thermo Fisher Scientific, Waltham, MA, USA) to evaluate the presence of DNA bands of the correct size. The size of the amplicon is specified for each primer set in Supplementary Table S1. Five microliters of the amplicons were then cleaned up with 2 µL of ExoSAP-IT™ PCR Product Clean-up Reagent (Applied Biosystems™), with the following thermal cycles: 37 °C for 4 min, 80 °C for 1 min and 4 °C for endless time. The sequencing reaction was performed with the same primers used for PCR, using BigDye Terminator v3.1 Cycle Sequencing kit (LifeTechnologies, Hong Kong, China) in a final volume of 10 µL as per manufacturer’s instructions. The thermal cycling conditions were 1 cycle at 96 °C for 45 s, followed by 28 cycles at 96 °C for 10 s and 50 °C for 5 s, 60 °C for 2 min. The sequencing products were purified using the Optima DTR 96-well plate (Resnova, London, UK) and analyzed in both directions using SeqStudio Genetic Analyzer (Applied Biosystems™). Sequences were aligned using MEGA version 6 [38] and compared with those available in GenBank using the Basic Local Alignment Search Tool (BLAST http://blast.ncbi.nlm.nih.gov/Blast.cgi (last accessed on 24 March 2025)). We assigned the species when the sequence identity in BLAST was ≥99%. In Supplementary Table S2, the accession numbers of the sequences matching with those we produced are reported.

The presence of TBEV was investigated using the alphaCube TBE kit^®^ (Mikrogen Diagnostik, Neuried, Germany), following the manufacturer’s instructions: 15.8 µL PCR mix, 0.2 µL RT enzyme and 4 µL of sample. Tick-specific mitochondrial *16S rRNA* was used for extraction and amplification quality control. Thirteen samples were not analyzed for the presence of TBEV due to insufficient material. All the real-time PCR runs were performed using the CFX96 thermal cycler (BioRad, Milan, Italy) under the following amplification conditions: 45 °C for 20 min, 95 °C for 5 min, followed by 45 cycles of 95 °C for 10 s, 60 °C for 20 s and a final extension step of 72 °C for 10 s.

## 3. Results

**Figure 1:**
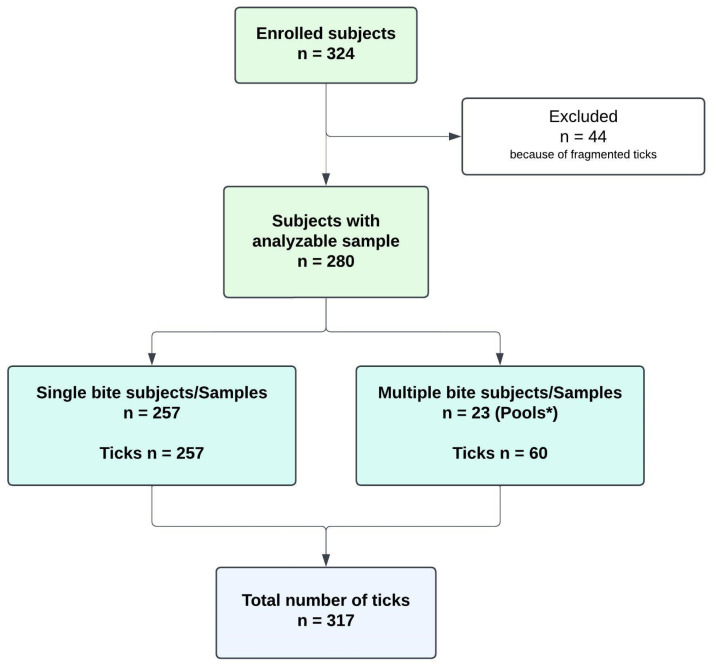
Flowchart of analyzed ticks. * For information about ticks’ pools, see Supplementary Table S3.

Ticks were collected from 324 participants. However, 44 ticks were excluded from microscopic analysis due to fragmentation, which prevented identification. As a result, the final analysis included 280 participants and 317 ticks (Figure 1). Among these 280 participants, 23 (8.2%) presented multiple bites, with up to nine ticks per person.

Of the 317 ticks analyzed microscopically, the majority belonged to *Ixodes* spp. (n = 303, 95.6%), while *Rhipicephalus* spp. (n = 2, 0.6%) and *Dermacentor* spp. (n = 1, 0.3%) were less common. For 11 ticks (3.5%), key structures were either missing or deformed, making it impossible to accurately match them with established dichotomous keys; so, these 11 ticks could not be classified but were anyway analyzed for the presence of TBPs. Regarding life stages, 195 (61.5%) were immature, including 180 nymphs (56.8%) and 15 larvae (4.7%). The remaining 121 (38.2%) were adult ticks, while one (0.3%) remained unclassified. Among the 121 adult ticks, 116 were females (95.9%), and 5 were males (4.1%).

Molecular assays were performed on 280 samples (257 single ticks and 23 ticks’ pools, Figure 1). Of these, 43 ticks (15.4%) carried at least one pathogen, with most detections occurring in the nymph and adult stages (Table 1). The identified pathogens included *Borrelia* spp. (n = 22), comprising 2 unidentified *Borrelia* spp., 8 *B. afzelii*, 6 *B. miyamotoi*, 2 *B. valaisiana*, 2 *B. garinii*, 1 *B. burgdorferi* s.s., 1 *B. spielmanii*. *Rickettsia* spp. was detected in 20 samples, including 2 unidentified *Rickettsia* spp., 11 *R. helvetica*, and 7 *R. monacensis*. Additionally, *Anaplasma phagocytophilum* was found in 5 and *Babesia venatorum* in 2 samples. Species identification was not feasible for some *Borrelia* and *Rickettsia* spp. due to insufficient DNA. Among the 280 samples, 13 had insufficient RNA for TBEV testing. Of the remaining 267 ticks, only 1 (0.4%) tested positive for TBEV.

Among the analyzed samples, the following main pathogens were detected: 22 samples (7.8%) were positive for *Borrelia*, 20 samples (7.1%) for *Rickettsia*, 5 (1.8%) for *Anaplasma phagocytophilum*, 2 (0.7%) for *Babesia venatorum*, and 1 sample (0.4%) for TBEV. Table 1 shows the data for each detected species.

Co-infections were identified in 6 out of 280 samples (2.1%), with most (5 out of 6) occurring in the *Ixodidae* nymphal stage. Two nymphal-stage samples carried *A. phagocytophilum* and *B. miyamotoi*, while another nymphal-stage sample harbored *R. monacensis* and *B. miyamotoi*. Additionally, *R. helvetica* and *B. afzelii* were detected in 1 nymph, and another nymph carried *Rickettsia* spp. and *B. venatorum*. A single case of triple infection was found in a combination of 1 adult and 1 nymphal-stage tick, harboring *B. miyamotoi*, *B. afzelii* and TBEV (Table 1).

**Figure 2:**
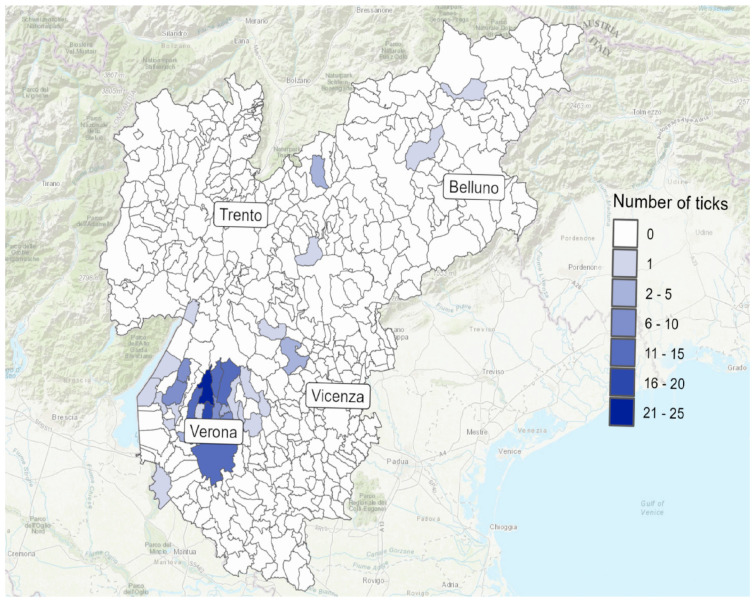
Veneto region map, showing the distribution of ticks collected in our study, for which the municipality of origin was known. The number of ticks collected for each municipality is indicated by the color scale.

**Figure 3:**
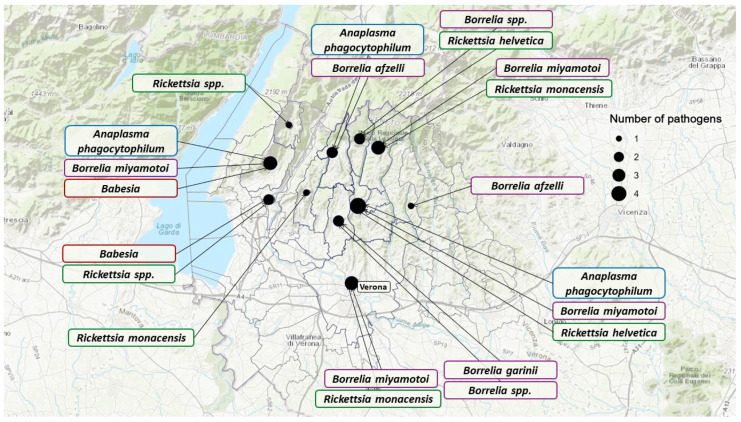
Veneto region map showing the distribution of pathogens identified in the samples for which the municipality of origin was known. TBEV was not represented in this figure since the municipality of tick origin was unknown.

Only 166 of the 280 subjects (59.3%) provided the likely location of their tick bite, accounting for geographic data on 177 ticks. Among these, 134 were from the Veneto region, with 129 specifically from the province of Verona. The geographic distribution of analyzed ticks and detected pathogens is shown in Figure 2 and Figure 3.

## 4. Discussion

This study provides new insights into the distribution of tick species and TBPs in the province of Verona. *Ixodes* spp. was the predominant tick species, reaffirming its role as a primary vector in Northern Italy. Notably, 61.5% of the analyzed ticks were in immature stages (nymphs and larvae), a higher proportion than the 42% reported for the same region in 2016 [15]. However, this percentage remains significantly lower than the nearly 90% observed in Austria and the province of Belluno, where ticks were collected either by dragging or following human bites [8,39]. This discrepancy may reflect differences in public awareness and the ability to detect smaller ticks in Verona. Enhancing public education campaigns is essential to improve tick identification and promote early removal, which is critical in reducing pathogen transmission risk, as the likelihood of infection increases with prolonged tick attachment [40].

The prevalence of TBPs identified in this study (15.4%) closely aligns with the 16% reported in a previous study from the same area [15], but compared to the previous study, more pathogen species were detected here. *Borrelia* spp. and *Rickettsia* spp. were the most frequently detected pathogens, exhibiting notable diversity within these genera and including the presence of emerging TBPs. Actually, in addition to the commonly known *B. afzelii*, *B. garinii*, and *B. burgdorferi* s.s., we identified *B. valaisiana*, *B. spielmanii*, and *B. miyamotoi.* While *B. spielmanii* and *B. valaisiana* have been associated with cutaneous forms of LB [17,41], the pathogenic role of *B. valaisiana* remains controversial [42]. The detection of *B. miyamotoi* in 2% of samples is particularly noteworthy, as this emerging pathogen, responsible for relapsing fever, has not previously been reported in ticks removed from humans in this region. Its nonspecific symptoms, including fever, fatigue, headache, and myalgia, complicate clinical recognition and require specific diagnostic tools that are often unavailable [43]. However, this study did not include follow-up data to assess symptom onset, highlighting the need for prospective studies monitoring clinical outcomes in tick-bitten individuals. The detection of emerging TBPs, such as *B. miyamotoi*, underscores the importance of enhancing public health surveillance and clinician awareness to improve early diagnosis and treatment strategies.

Among Rickettsia species, *R. helvetica* and *R. monacensis* were detected in 3.9% and 2.5% of ticks, respectively, data consistent with previous reports from northeastern Italy [15,44]. Although *R. helvetica* is widespread across Europe and has been suspected to cause mild illnesses such as fever and myalgia, only three human cases have been documented in Italy [45]. Similarly, *R. monacensis* has been associated with Mediterranean spotted fever-like illnesses, including a case reported in Sardinia in 2011 [46]. Despite their relatively frequent detection, the pathogenicity and clinical impact of these rickettsiae remain poorly understood, highlighting the need for further research and improved diagnostic tools for human diagnosis. Additionally, the presence of *A. phagocytophilum* and *B. venatorum* highlights the regional TBP diversity. While *A. phagocytophilum* infection has been documented in northeastern Italy [15], human cases remain rare and likely underdiagnosed due to nonspecific symptoms and limited access to diagnostic tests [47]. Similarly, *B. venatorum*, known to cause babesiosis in immunocompromised individuals, is frequently identified in ticks but rarely in human cases in Italy, with the only documented case reported in 2004 [48]. The discrepancy suggests that babesiosis may be underdiagnosed, especially in mild or asymptomatic infections.

This study is the first to report the presence of the TBE virus in a tick collected from a human in Verona province. While most TBEV infections are asymptomatic or present with mild symptoms, approximately 10% of cases develop neurological complications, with sequelae rates of 26–46% and a fatality rate of 1% [49,50]. As no specific antiviral treatment is available, vaccination remains the most effective preventive strategy. However, low vaccination coverage in the region, likely due to the lack of routine inclusion in immunization programs and limited public awareness, underscores the urgent need for targeted public health initiatives.

Co-infections, although relatively rare, complicate clinical presentation and diagnosis. Overlapping symptoms can obscure the identification of individual pathogens, increasing the risk of misdiagnosis and inappropriate treatment [8,15]. These findings highlight the importance of improving diagnostic capabilities and increasing clinical awareness to address the risks associated with multiple TBPs.

This study offers valuable epidemiological insights but has several limitations. The absence of clinical data from patients bitten by infected ticks restricts our ability to assess the clinical relevance of TBPs, particularly emerging pathogens. A larger, prospective study incorporating clinical data, molecular analysis, and repeated serological testing would provide deeper insights into human infection rates and symptom correlations. Additionally, reliance on patient recall to determine the likely location of tick bites introduces potential bias, highlighting the need for more systematic geographic data collection. The restricted panel of TBPs tested may have underestimated pathogen diversity, as rarer pathogens, such as *R. slovaca* and *Neoehrlichia mikurensis*, were not included. Furthermore, the use of pooled samples for molecular analysis in cases of multiple tick bites may have led to an underestimation of individual pathogen infection rates.

Despite these limitations, this study enhances understanding of TBP circulation in Verona and highlights the need for improved surveillance, diagnostics, and public health interventions. Integrating tick surveillance in the natural habitat with epidemiological studies is essential for assessing TBP risks, improving detection, and guiding preventive strategies. Increased awareness among healthcare professionals and the public is crucial for reducing the burden of TBDs and improving response measures.

## 5. Conclusions

This study describes the presence of multiple TBPs in the province of Verona, a region where only LB and TBE have been reported in humans. These findings highlight the need for enhanced tick surveillance in a one-health approach to better assess the potential health risks to the local population. Raising awareness among the public and healthcare professionals is essential to improve preventive measures, including promoting TBE vaccination. Public health initiatives should emphasize tick bite prevention, early removal, and vaccination, particularly in areas where TBD remains under-recognized.

Future research should integrate microbiological findings from ticks with clinical, serological, and microbiological data from humans. Such a prospective study would provide crucial insights into the clinical relevance of emerging TBPs, bridging the gap between pathogen detection in vectors and their impact on human health. This comprehensive approach would support more effective prevention, diagnosis, and management of TBDs in the region.

## Figures and Tables

**Table 1:** Results of TBP detection in the analyzed samples. Tick species and life stages in which pathogens were detected are also reported.

	n/N (%)	Species	Stage
*Borrelia afzeli*	8/280 (2.9)	All *Ixodes* spp.	3 adults, 2 nymphs, 3 pool (n. 7, 8, 10)
*Borrelia miyamotoi*	6/280 (2.1)	5 *Ixodes* spp., 1 non-identifiable	2 adults, 3 nymphs, 1 pool (n. 10)
*Borrelia* spp.	2/280 (0.7)	All *Ixodes* spp.	2 adults
*Borrelia garinii*	2/280 (0.7)	All *Ixodes* spp.	1 adult, 1 nymph
*Borrelia valaisiana*	2/280 (0.7)	All *Ixodes* spp.	1 adult, 1 nymph
*Borrelia burgdorferi* s.s.	1/280 (0.4)	All *Ixodes* spp.	1 adult
*Borrelia spielmanii*	1/280 (0.4)	All *Ixodes* spp.	1 nymph
*Rickettsia helvetica*	11/280 (3.9)	All *Ixodes* spp.	7 adults, 3 nymphs, 1 pool (n. 7)
*Rickettsia monacensis*	7/280 (2.5)	All *Ixodes* spp.	1 adult, 6 nymphs
*Rickettsia* spp.	2/280 (0.7)	All *Ixodes* spp.	2 nymphs
*Anaplasma phagocytophilum*	5/280 (1.8)	All *Ixodes* spp.	2 adults, 3 nymphs
*Babesia venatorum*	2/280 (0.7)	All *Ixodes* spp.	2 nymphs
*Tick-borne encephalitis virus*	1/267 * (0.4)	All *Ixodes* spp.	1 pool (n. 10)

N is the number of samples tested. Data are expressed as n/N (%). * 13 samples had insufficient RNA left for testing.

## Data Availability

The original contributions presented in this study are included in the article/Supplementary Material. Further inquiries can be directed to the corresponding author.

## Supplementary Materials

The following supporting information can be downloaded at https://www.mdpi.com/article/10.3390/microorganisms13050965/s1: Table S1. Primers and probe sequences used for the in-house real-time and end-point PCR assays; Table S2. GenBank (NCBI) accession number of the sequences matching with those we produced; Table S3: Details of the 23 pools.
