# Supplementary material for: Surveillance of Tick-Borne Pathogens in Ticks from Humans in the Province of Verona, Italy (2018–2022): A Prospective Study
Source: Microorganisms. 2025 Apr 23;13(5):965. doi: 10.3390/microorganisms13050965 (PMC12114187; doi:10.3390/microorganisms13050965)
Supplement: Supplementary file 1 [file microorganisms-13-00965-s001.zip › microorganisms-3540718-supplementary.pdf]

Supplementary Tables

Table S1. Primers and probes sequences used for the in-house real-time and end-point PCR assays. For the end-point PCR assays, the expected amplicon size is reported.

Pathogen	Target gene	Primers	Sequence 5'-3'	Amplicon size
Borrelia spp.	*23S rRNA*	Bb23s_F	CGAGTCTTAAAAGGGCGATTTAGT	NA
		Bb23s_R	GCTTCAGCCTGGCCATAAATAG	
		Bb23s _probe	FAM-AGATGTGGTAGACCCGAAGCCGAGTG-BHQ1	
Borrelia miyamotoi	*p4*	Bmp41F	TTGCTTGTGCAATCATAGCC	NA
		Bmp41R	GCAAATCTTGGTGCTTTTCAA	
		Bmp41S	Cy5-AGATGCCACAATTTCATCTGTCATTA-BBQ-650	
Borrelia genospecies	*flaB*	FBsppnew	TGAGWWGGYGCTGTAGCAG	NA
		Rbsppnew	CAAGATGARGCDATTGCTGTAAA	
B. afzelii probe	*flaB*	P_Bafz	ROX-TTCTTGAGCACCCTCTTGAACAGG -BHQ2	
B. garinii probe	*flaB*	P_Bgar	Cy5 –CTTGTTGAGCTCCTTCTTGAACAGG–BHQ2	
B. burgdorferi sensu stricto probe	*flaB*	P_Bbbs	FAM –TCCTTCCTGTTGAACACCCTCTTG–BHQ1	
Anaplasma phagocytophilum	*msp2*	ApMSP2f	ATGGAAGGTAGTGTTGGTTATGGTATT	NA
		ApMSP2r	TTGGTCTTGAAGCGCTCGTA	
		ApMSP2p	HEX -TGGTGCCAGGGTTGAGCTTGAGATTG-TAMRA	
Rickettsia spp.	*rompB*	RompB OFm	GTAACCGGAARTAATCGTTTCGT	511 bp
		RompB ORm	GCTTTATAACCAGCTAAACCRCC	
Borrelia spp.	*fla*	FLA1	AGAGCAACTTACAGACGAAATTAAT	482 bp
		FLA2	CAAGTCTATTTTGGAAAGCACCTAA	
Babesia spp.	*18SrRNA*	BJ1	GTCTTGTAATTGGAATGATGG	500 bp
		BN2	TAGTTTATGGTTAGGACTACG	

rompB = outer-membrane protein gene; *fla* = flagellin gene; NA = not applicable.

Table S2: GenBank (NCBI) accession number of the sequences matching with those we produced. The percent of sequence identity with our Sanger sequences is reported.

ID	Specie	PERCENT IDENTITY
KJ663750	*Rickettsia helvetica*	100%
EU883092	*Rickettsia monacensis*	100%
OK598971	*Babesia venatorum*	99%
KU672550	*Borrelia spielmanii*	100%
CP009117	*Borrelia valaisiana*	100%

Table S3. Details of the 23 pools.

ID	Genus	Detail of ticks collected	Number of ticks
Pool1	*Ixodidae*	2 nymphs	2
Pool2	*Ixodidae*	2 nymphs	2
Pool3	*Ixodidae*	2 nymphs	2
Pool4	*Ixodidae*	2 larvae	2
Pool5	*Ixodidae*	1 adult female, 8 nymphs	9
Pool6	*Ixodidae*	4 nymphs	4
Pool7	*Ixodidae*	4 nymphs	4
Pool8	*Ixodidae*	2 nymphs	2
Pool9	*Ixodidae*	2 nymphs	2
Pool10	*Ixodidae*	1 adult female, 1 nymph	2
Pool11	*Ixodidae*	2 adult females	2
Pool12	*Ixodidae*	2 nymphs	2
Pool13	*Ixodidae*	2 larvae	2
Pool14	*Ixodidae*	1 nymph, 1 larva	2
Pool15	*Ixodidae*	2 nymphs	2
Pool16	*Ixodidae*	1 adult female, 1 nymph	2
Pool17	Non identifiable	1 adult, 1 nymph	2
Pool18	*Ixodidae*	4 nymphs	4
Pool19	*Ixodidae*	2 nymphs	2
Pool20	*Ixodidae*	2 nymphs	2
Pool21	*Ixodidae*	2 nymphs	2
Pool22	*Ixodidae*	2 nymphs	2
Pool23	*Ixodidae*	3 nymphs	3
